# An Interesting Case of COVID-19 Induced Reversed Takotsubo Cardiomyopathy and Insight on Cardiac Biomarkers

**DOI:** 10.7759/cureus.11296

**Published:** 2020-11-02

**Authors:** Ankur Panchal, Andreas Kyvernitakis, Robert Biederman

**Affiliations:** 1 Internal Medicine, University of Pittsburgh Medical Center, Pittsburgh, USA; 2 Cardiovascular Disease, Allegheny Health Network, Pittsburgh, USA

**Keywords:** troponin, myocarditis, covid-19, imaging, electrocardiogram, cardiomyopathy

## Abstract

Cardiovascular involvement is common in COVID-19 patients and is associated with increased mortality, especially in patients with pre-existing cardiac comorbidities. Elevated levels of troponin have been noted to predict worse prognosis for COVID-19 patients, regardless the physiology of insult. We report a case of a 65-year old man who was admitted for acute hypoxemic respiratory failure due to COVID-19 disease that rapidly decompensated and required mechanical ventilation. He responded well with medical treatment and was successfully extubated. Interestingly, his serum troponin T levels remained negative (<0.01 ng/mL) until day 10, when it was noted to be elevated despite him being completely asymptomatic. Echocardiogram revealed new left ventricular wall motion abnormalities suggestive of reverse Takotsubo cardiomyopathy. Unfortunately, he suffered from a pulseless electrical arrest less than 24 hours later and eventually expired. This case shows that a policy of trending troponin levels may be valuable as a screening tool for critically ill COVID-19 patients and may be beneficial for early silent validation of cardiovascular involvement in these patients, who could otherwise be asymptomatic yet presage adverse clinical events. Moreover, using troponin as a screening tool may lead to decreased utilization of echocardiography and reduce the exposure of COVID-19 to healthcare workers.

## Introduction

Coronaviruses are single positive-stranded RNA viruses and known to cause acute respiratory distress syndrome [1]. Severe acute respiratory syndrome coronavirus - 2 (SARS-CoV-2) is a novel coronavirus, first recognized in a recent outbreak in Wuhan, China and propagated across the globe rapidly. The World Health Organization declared it to be a pandemic in March 2020 [2]. COVID-19 has challenged the medical field requiring arduous efforts to conquer this novel disease. To date, over 27 million people are affected, resulting in over 850,000 deaths. Cardiovascular involvement such as myocarditis, acute coronary syndrome, and conduction abnormalities are commonly reported in hospitalized COVID-19 patients and associated with increased mortality, especially in patients with pre-existing cardiac comorbidities [3]. We hereby present an interesting case of COVID-19 related reverse Takotsubo Cardiomyopathy with insight on cardiac biomarkers.

## Case presentation

**Figure 1:**
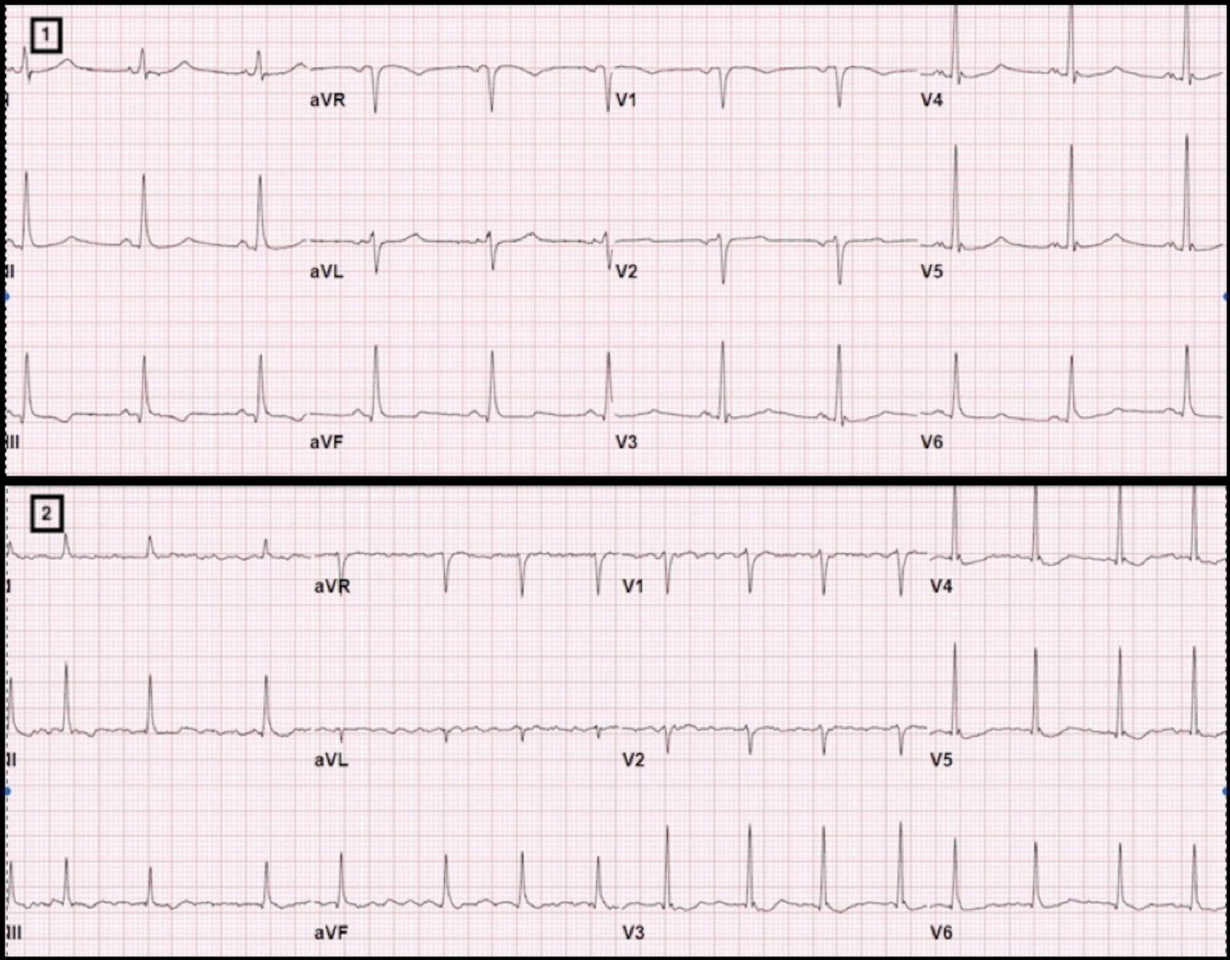
Electrocardiograms on presentation and just before the patient’s pulseless electrical arrest showing new non-specific T wave abnormalities on the anterolateral leads (images 1 and 2). Patient had known paroxysmal atrial fibrillation.

A 65-year-old pleasant gentleman presented to emergency department of our hospital complaining of malaise, shortness of breath and dry cough for 2 weeks. His past medical history was notable for hypertension, diabetes and paroxysmal atrial fibrillation on systemic anticoagulation. He denied having any anginal symptoms. Initial vitals were significant for blood pressure of 159/70 mmHg, respiratory rate of 23/min, temperature of 102.8 º Fahrenheit with oxygen saturation of 87% on 6L oxygen via nasal cannula. Chest x-ray was suggestive of multifocal pneumonia and initial electrocardiogram (ECG) showed normal sinus rhythm without any significant ST-T wave abnormalities (Figure 1). He was noted to be progressively worsening over the next 24 hours resulting in sudden hemodynamic decompensation requiring ventilator support and vasopressors. Other pertinent lab results were ferritin 804 ng/mL, D-dimer 0.91 mcg/mL, C-reactive protein (CRP) 12.6 mg/L and normal troponin T (<0.01 ng/mL). He was found to be positive for SARS-CoV-2 through PCR testing and negative for influenza virus.

**Figure 2:**
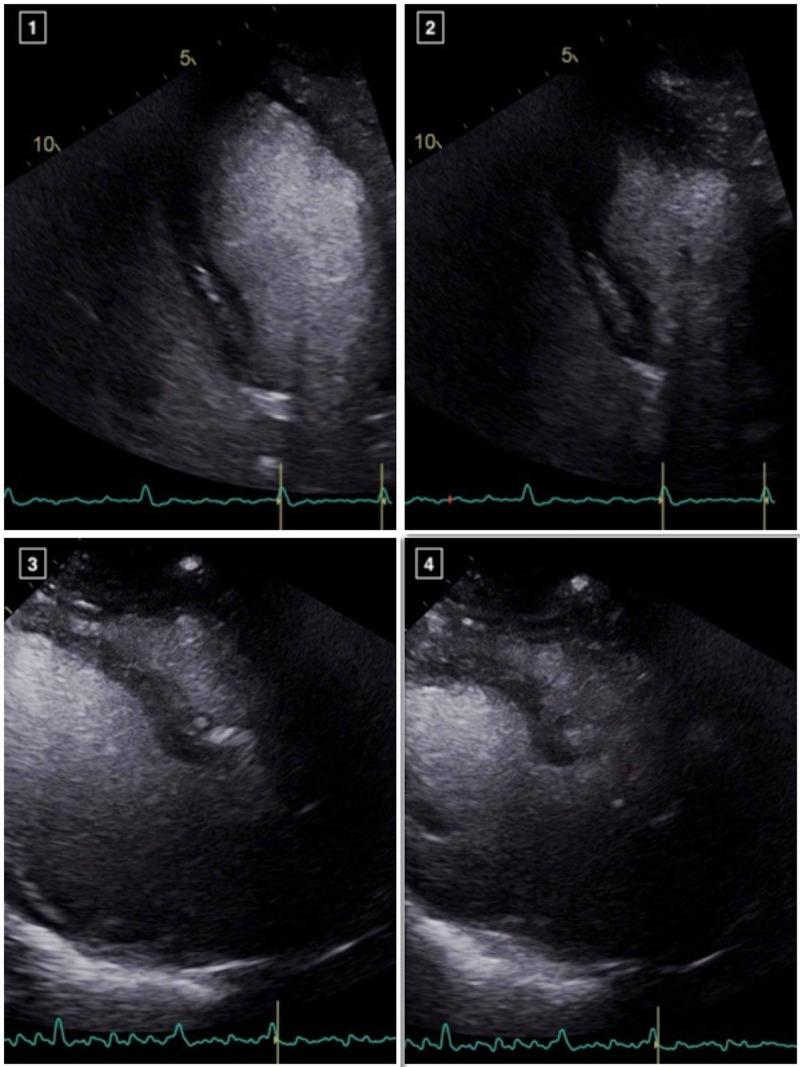
Apical 4-chamber views and parasternal long axis views demonstrating basal to mid LV systolic dysfunction with preserved distal and apical segments, in a reverse TakoTsubo pattern (images 1-4).

He received azithromycin, hydroxychloroquine and plasmapheresis, with good response and was eventually successful extubated. His blood cultures remained negative and transthoracic echocardiogram (TTE) checked during his critical illness was noted to demonstrate normal biventricular functions without any conduction abnormalities. As a part of our institutional protocol, daily troponin T were checked and remained negative (<0.01 ng/mL) until day 10, when it was noted to be elevated (peak of 0.61 ng/mL). Troponin elevation triggered an ECG which showed nonspecific ST-T wave abnormalities (QTc 420 ms, QTc 439 ms on admission) and TTE performed on the same day revealed new left ventricular (LV) regional wall motion abnormalities with hypokinesis of the basal to mid segments and hyperkinetic apical segments, suggestive of a reverse Takotsubo cardiomyopathy, likely in the setting of COVID-19 myocarditis (Figure 2). Cardiac catheterization was considered, but given the patient's presentation, imaging and electrical data before and after the troponin elevation, ischemic heart disease was unlikely. Therefore cardiac catheterization was not performed to further limit the exposure to COVID-19. He was on 2L oxygen via nasal cannula but otherwise completely asymptomatic and denied having any anginal or cardiac symptoms. Unfortunately, he suffered pulseless electrical arrest 24 hour later with successful return of spontaneous circulation though comfort measure was pursued due to worsening clinical condition with development of multiorgan failure and eventually expired. Cardiac MRI was not performed due to acuity and rapid decompensation of the patient's clinical condition after the diagnosis of reverse Takotsubo Cardiomyopathy. 

## Discussion

Cardiovascular involvement in COVID-19 is due to various reported mechanisms including severe inflammatory response and cytokine storm, plaque destabilization due to oxidative stress, respiratory failure causing oxygen supply-demand mismatch and possible direct myocardial involvement [4]. Most of the published literature to date has focused on identifying cardiac manifestations and the underlying mechanisms of COVID-19. Elevated troponin levels have been noted to predict worse prognosis for COVID-19 patients, regardless the physiology of insult [5]. Unfortunately, not much is known on the utility of using cardiac biomarkers as a screening tool for cardiac involvement in these patients. Previous studies have reported that up to 28% of hospitalized COVID patients have elevated troponin levels and those patients have increased likelihood for requiring mechanical ventilation with up to 60% mortality in patients with cardiac comorbidities [6, 7]. Similar to our patient, there are a few cases of Takotsubo cardiomyopathy and one of reverse Takotsubo cardiomyopathy in the setting of COVID-19 hospitalized patients reported previously [8-10]. Moreover, emerging literature shows higher mortality in COVID-19 related Takotsubo Cardiomyopathy compared to Takotsubo Cardiomyopathy not related to COVID-19 [11, 12].

Although our patient responded well to initial medical treatment and was at the edge of recovery, a sudden rise in troponin was noted that was associated with new LV wall motion abnormalities. Since our patient was asymptomatic, we would not have otherwise identified the cardiac involvement of COVID-19. Taking this into account, we propose that daily troponin levels can be used as a screening tool of cardiac involvement in COVID-19 patients and have the potential to result in early detection and prompt therapeutic decisions, especially with emerging therapies available for hospitalized COVID-19 patients. Moreover, several studies have shown that any troponin elevation in this population portends worse outcomes. Finally, including troponin levels in routine blood draws in these patients will assist in the utilization of echocardiography and limit the exposure to this highly contagious virus.

## Conclusions

Cardiovascular involvement in COVID-19 is associated with increased morbidity and mortality and has thus become a cardinal focus in the early stage of this evolving novel virus. Takotsubo Cardiomyopathy is a rare manifestation of COVID-19 disease and is associated with higher mortality. We hereby report an interesting case of COVID-19 related reverse Takotsubo cardiomyopathy with insight on cardiac biomarkers as they may play an important role in early silent validation of cardiovascular insult in otherwise asymptomatic patients, though more research is warranted in this area. 
